# Characterization of N^6^-methyladenosine in cattle-yak testis tissue

**DOI:** 10.3389/fvets.2022.971515

**Published:** 2022-08-09

**Authors:** Xingdong Wang, Jie Pei, Shaoke Guo, Mengli Cao, Yandong Kang, Lin Xiong, Yongfu La, Pengjia Bao, Chunnian Liang, Ping Yan, Xian Guo

**Affiliations:** ^1^Key Laboratory of Yak Breeding Engineering of Gansu Province, Lanzhou Institute of Husbandry and Pharmaceutical Sciences, Chinese Academy of Agricultural Sciences, Lanzhou, China; ^2^Key Laboratory of Animal Genetics and Breeding on Tibetan Plateau, Ministry of Agriculture and Rural Affairs, Lanzhou, China

**Keywords:** cattle-yak, male sterility, spermatogenesis, N^6^-methyladenosine, testicular tissue

## Abstract

N^6^-methyladenosine (m^6^A) is the most common form of eukaryotic mRNA modification, and it has been shown to exhibit broad regulatory activity in yeast, plants, and mammals. The specific role of m^6^A methylation as a regulator of spermatogenesis, however, has yet to be established. In this experiment, through a series of preliminary studies and methylated RNA immunoprecipitation sequencing, the m^6^A map of cattle-yak testicular tissue was established as a means of exploring how m^6^A modification affects cattle-yak male infertility. Cattle-yak testis tissues used in this study were found to contain sertoli cells and spermatogonia. Relative to sexually mature yak samples, those isolated from cattle-yak testis exhibited slightly reduced levels of overall methylation, although these levels were significantly higher than those in samples from pre-sexually mature yaks. Annotation analyses revealed that differentially methylated peaks were most concentrated in exonic regions, with progressively lower levels of concentration in the 3'-untranslated region (UTR) and 5'-UTR regions. To further explore the role of such m^6^A modification, enrichment analyses were performed on differentially methylated and differentially expressed genes in these samples. For the cattle-yaks vs. 18-months-old yaks group comparisons, differentially methylated genes were found to be associated with spermatogenesis-related GO terms related to the cytoskeleton and actin-binding, as well as with KEGG terms related to the regulation of the actin cytoskeleton and the MAPK signaling pathway. Similarly, enrichment analyses performed for the cattle-yaks vs. 5-years-old yaks comparison revealed differentially methylated genes to be associated with GO terms related to protein ubiquitination, ubiquitin ligase complexes, ubiquitin-dependent protein catabolism, and endocytotic activity, as well as with KEGG terms related to apoptosis and the Fanconi anemia pathway. Overall, enrichment analyses for the cattle-yaks vs. 18-months-old yaks comparison were primarily associated with spermatogenesis, whereas those for the cattle-yaks vs. 5-years-old yaks comparison were primarily associated with apoptosis.

## Introduction

The yak (*Bos grunniens*) is a highly recognizable symbol of the Qinghai-Tibet Plateau (QTP) and nearby high-altitude regions where it is endemic (1, 2), primarily residing at altitudes of 2,500–6,000 m (3). Yaks are well-suited to surviving in the harsh climate of the QTP, and can readily tolerate environmental stressors including hypoxia, cold, and local diseases (3), serving as the primary form of livestock for humans living in this area (4). Highlanders use yaks both as pack animals and as a source of meat, milk, fuel, and fur (5). They are also central components of efforts to conserve agrobiodiversity, promote socio-economic development, and maintain rangeland ecosystems in these plateau regions (6). Relative to other cattle species, however, yaks exhibit lower rates of growth, reduced production performance (7), lower levels of fertility, and late sexual maturity (8). In most cases, yaks present with short-lived, relatively inconspicuous estrus behaviors beginning at 3–4 years of age, producing just once every 2 years or twice every 3 years (9).

Interspecific hybridization can play a central role in the adaptation and evolution of particular species in nature, with heterosis often resulting from the hybridization of genetically distinct animals (10). In an effort to improve yak production performance, researchers and breeders have sought to combine the excellent productivity of other cattle species with the QTP-adapted traits of yaks, such as cold tolerance, through interspecific hybridization (11). The resultant cattle-yaks, which are a hybrid of yaks and conventional cattle (*Bos taurus*), possess the high productivity of cattle and the adaptability of yaks to high-altitude conditions (12). Cattle-yaks exhibit excellent heterosis with respect to growth speed, disease resistance, drought tolerance, meat quality, and adaptability to the plateau environment (13, 14). Meat from these cattle-yak hybrids, which are larger than yaks, contains higher levels of protein and lower fat content as compared to yak meat, thus fulfilling a need for the production of healthier high-quality foods fit for public consumption (15). However, this heterosis cannot be effectively leveraged owing to the fact that F1 male cattle-yaks exhibit an inability to produce sperm, resulting in sterility (16). Despite the normal development of external cattle-yak reproductive organs, spermatogenesis in these hybrids is blocked after the primary spermatocyte stage, with only a relatively small number of autosomes from a few spermatocytes being evident in the meiotic synaptonemal complex (SC) in these animals (17). Prior research efforts have leveraged multifunctional strategies including transcriptomic, proteomic, genetic, physiological, and endocrinological approaches in an effort to clarify the mechanistic basis for male hybrid sterility (12). However, the mechanisms that ultimately drive male cattle-yak infertility have yet to be firmly established.

Spermatogenesis is a complex process wherein diploid spermatogonial stem cells (SSCs) undergo differentiation to produce haploid spermatozoa through tightly regulated mitotic, meiotic, and spermatogenic processes that are controlled at the transcriptional, post-transcriptional, and translational levels (18). Epigenetic regulation can also shape the development of the male reproductive system, with both developmental and environmental factors influencing the establishment of epigenetic marks that, in turn, govern both the early stages of embryonic development and gametogenic processes (19). Several studies have shown that impaired epigenetic dysregulation can disrupt human spermatogenesis, contributing to male infertility and associated spermatogenesis disorders (20). Over 100 different chemical RNA modifications have been detected to date, including the N^1^-methyladenosine (m^l^A), m^6^A, and 5-methylcytosine (m^5^C) modifications (21). In eukaryotic cells, m^6^A is the most common form of RNA modification (22), controlling all stages of the RNA metabolism process including the folding, maturation, stabilization, and translation of modified mRNAs (23). Notably, m^6^A RNA modification has been shown to be crucial for male germ line development, particularly in the context of mammalian spermatogenesis (24, 25).

Analyses of testes tissue samples from a range of species have highlighted relationships between the process of spermatogenesis and RNA m^6^A modification (26–28). Male mice in which the *ALKBH5* gene has been knocked out exhibit increased m^6^A levels and impaired fertility as compared to wild-type (WT) littermates, suggesting a role for this m^6^A demethylase in this physiological setting (29, 30). Notably, *METTL3*-deficient murine embryonic stem cells do not undergo normal differentiation, and the mutation of *METTL3* is associated with embryonic lethality in mice (29). Two *FTO* mutations have been linked to reductions in semen quality, and the dysfunction of the FTO protein has been tied to decreased male fertility (31). Single-cell sequencing data from human testis samples have revealed the expression of RNA m^6^A regulatory genes in almost all testis cell types, including both somatic and spermatogenic cells (32). Consistently, knocking down *FTO, ALKBH5, METTL3, METTL14, YTHDF2*, or *YTHDC2* impairs normal gametogenesis and fertility (33). These prior results thus strongly suggest that altered RNA m^6^A modification may play a causative role in the regulatory processes underlying the molecular pathogenesis of male infertility.

The present study was developed with the goal of studying the mechanisms underlying m^6^A modification and associated regulatory processes in samples of testis tissue from sterile male cattle-yaks. To that end, a MeRIP-seq approach was used to establish whole transcriptomic m^6^A profiles for samples of testis tissue from normal sexually matured sterile male cattle-yaks. Using this approach, differentially methylated peaks were identified by comparing 5-years-old cattle-yaks (T group) with pre-sexually mature 18-months-old yaks (Y group) and post-sexually mature 5-years-old cattle-yaks (M group) that have been published previously (34), thus offering insight into the regulatory importance of m^6^A methylation in the context of cattle-yak sterility.

## Materials and methods

### Ethics statement

All animal-related procedures were consistent with guidelines established by the China Council on Animal Care and the Ministry of Agriculture of the People's Republic of China. The Animal Care and Use Committee of the Lanzhou Institute of Husbandry and Pharmaceutical Sciences Chinese Academy of Agricultural Sciences approved all yak handling procedures for this study (Permit No: SYXK-2014-0002).

### Tissue sample isolation

Samples of testicular tissue were harvested following castration from three normal 5-years-old cattle-yaks in Xiahe County, Gannan Tibetan Autonomous Prefecture (N34°51 ', E102°26 '). Prior to tissue sample collection, iodophor was used to disinfect the samples. After sample isolation, a surgical needle was used to suture the wound site, with penicillin/streptomycin then being administered to protect against infection. The white testicular membrane was removed, and tissues were rinsed using 1 × PBS. Samples were then minced into 5 cm^3^ segments and transferred to a cryotube, after which they were snap-frozen using liquid nitrogen. A subsample of these testicular tissue isolates was also immobilized in Bouin's Fluid (SolarBio, Beijing, China). Samples were transferred to Lanzhou Institute of Husbandry and Pharmaceutical Sciences, Chinese Academy of Agricultural Sciences for subsequent use immediately after collection.

### Hematoxylin and eosin staining

After fixation, testicular tissue samples were dehydrated using 75% ethanol, paraffin-embedded, and cut into 6 mm sections that were then stained with an improved H&E staining kit (SolarBio, Beijing, China) based on provided direction. Sections were then sealed using neutral gum and imaged with a Pannoramic 250 digital section scanner (Drnjier, Jinan, China).

### RNA isolation and CDNA preparation

TRIzol (Invitrogen, CA, USA) was used to extract RNA from tissue samples, after which the purity (OD260/280 ratio) and concentration of RNA in these samples was measured with a NanoDrop 2,000 instrument (ThermoFisher Scientific, MA, United States). RNA at a concentration of 500–5,000 ng/mL with an OD260/280 ratio of 1.9–2.1 was selected and diluted to 500 ng/mL. Then, cDNA was prepared using a Transcriptor First Strand cDNA Synthesis Kit (Takara Bio Inc., Dalian, China), after which it was stored at−80°C for subsequent use.

### qPCR

Analyses of cattle-yak testis m^6^A status were performed using appropriate primers (Supplementary Table S1), a LightCycler^®^ 96 Instrument (Roche, Beijing, China), and reaction conditions (Supplementary material) reported previously (34). Analyzed RNA methylation-associated genes detected *via* qPCR included *METTL3, METTL14, WTAP, FTO, ALKBH5, YTHDF1/2/3, YTHDC1/2, RBM15, VIRMA*, and *ZC3H13*. Relative gene expression levels were assessed *via* the 2^−^ΔΔCT method (35), with *GAPDH* serving as a normalization control. Analyses were repeated in triplicate, and differences in gene expression were compared *via* analyses of variance (ANOVAs).

### m^6^A content analyses

An EpiQuik RNA Methylation Quantification Kit (Epigentek, P-9005, NY, United States) was used to detect mRNA m^6^A levels in cattle-yak testis samples based on provided directions.

### MeRIP-seq and RNA-seq analyses

Sequencing libraries (Supplementary material) were prepared with the Illumina TrueSeq Stranded mRNA platform as in prior reports (34). The Illumina HiSeq X10 System at OE Biotech Co., Ltd. (Shanghai, China) was used to perform paired-end sequencing. All data were submitted to the Gene Expression Omnibus (GEO) database (Accession number: GSE205649). RNA-seq data were subjected to quality control and statistical analyses (Supplementary material) as in prior reports (34).

### Statistical analysis

SPSS v 21.0 was used for all statistical analyses (36). Data were compared *via* one-way ANOVAs, with *P* < 0.05 or *P* < 0.01 as the threshold of significance (37).

## Results

### Histopathological analyses of cattle-yak testicular tissue samples

**Figure 1:**
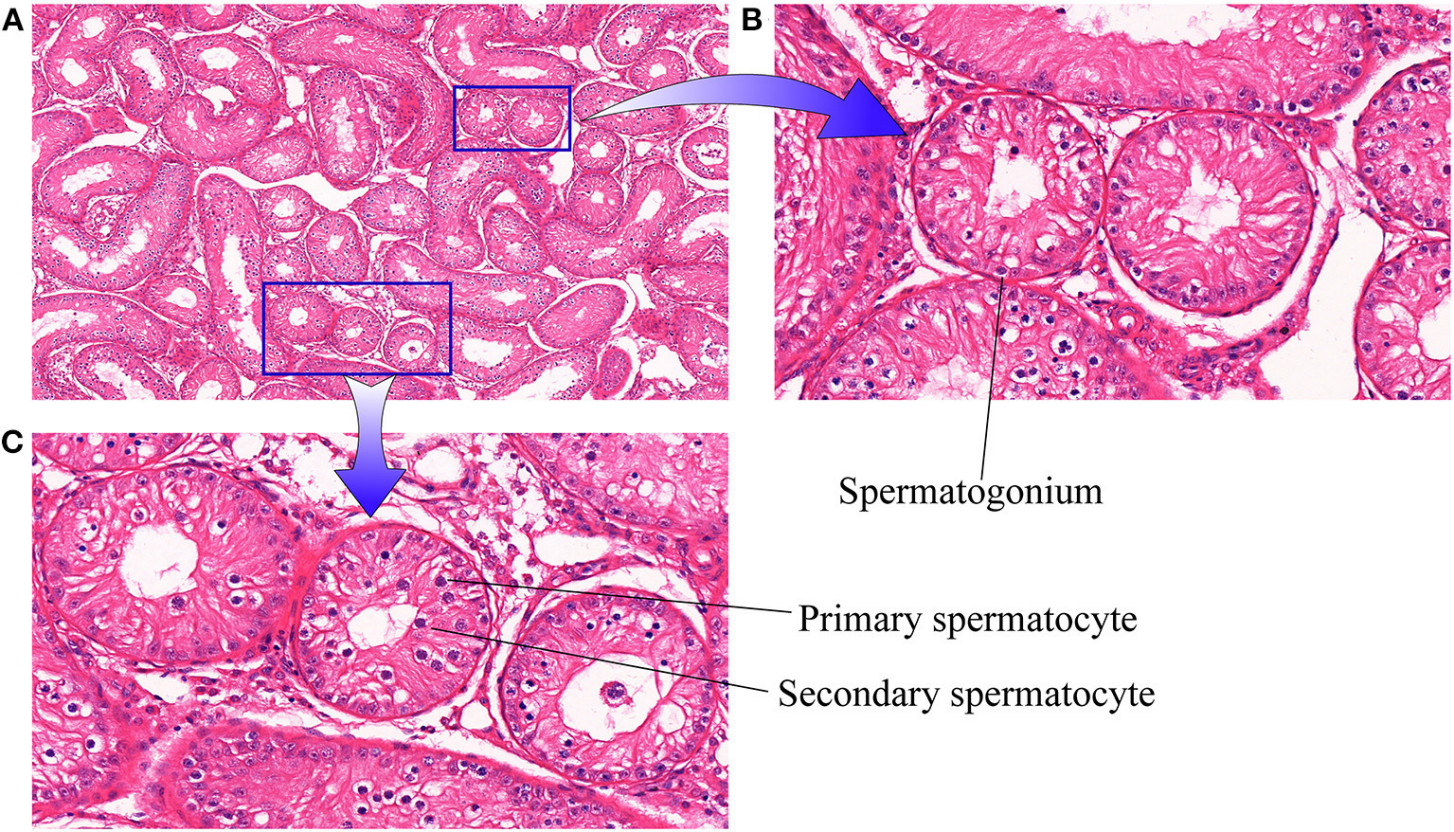
Cattle-yak testicular tissue sections. **(A)** 10× magnification; **(B,C)** 40× magnification.

Initially, histopathological examination of cattle-yak testicular tissue samples was performed under a light microscope (10x, Figure 1A). Visually, the seminiferous tubules were loosely arranged with air bubbles between them. When examined under further magnification (40×, Figures 1B,C) germ cells in these testis tissues were found to primarily be arranged in a spermatogonia monolayer attached to the seminiferous tubule basement membrane with only limited numbers of visible spermatocytes.

### qPCR and global m^6^A quantification analyses

**Figure 2:**
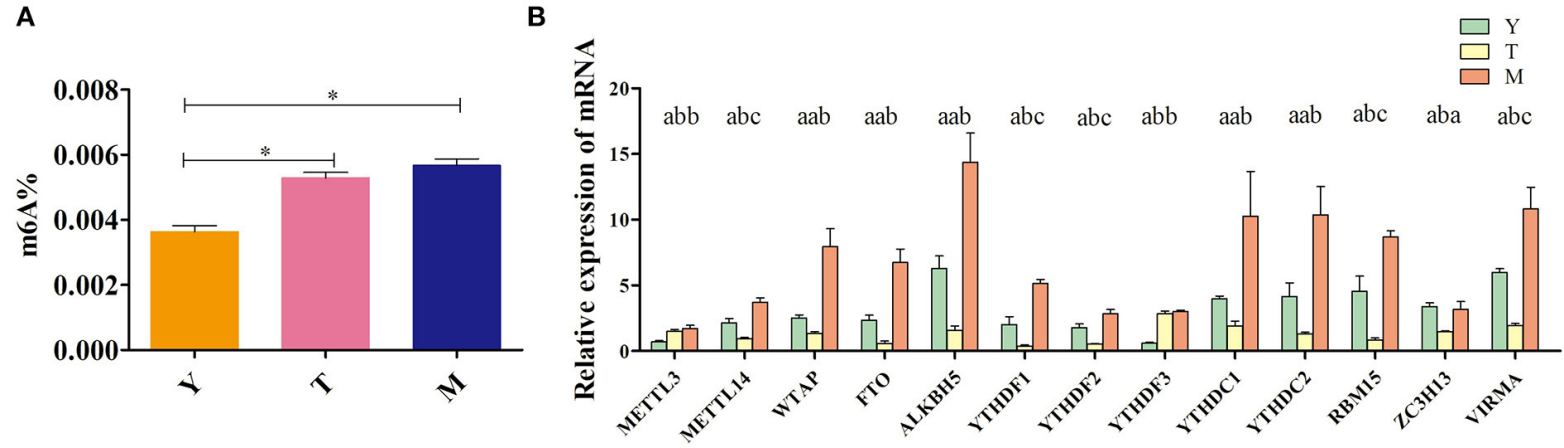
Analyses of m^6^A levels in yak and cattle-yak testicular tissues. **(A)** Overall levels of testis tissue methylation. Y: 18-months-old yak, T: cattle-yak, M: 5-years-old yak, **P < 0.01, *P < 0.05. **(B)** Methylation-related enzyme expression in the indicated yak groups. Different lowercase letters correspond to significant differences among groups (*P* < 0.05), whereas identical letters indicate a lack of any significant differences (*P* > 0.05).

Next, overall methylation levels were compared between testicular tissue samples collected from 5-years-old cattle-yaks, 18-months-old yaks (pre-sexually mature yaks), and 5-years-old yaks (post-sexually mature yaks). While the levels of methylation in 5-year-old yaks were higher than in 5-years-old cattle-yaks, these differences were not significant. However, methylation levels were significantly higher in testicular samples from both 5-years-old yaks and cattle-yaks in comparison to 18-months-old yaks (Figure 2A). The mRNA level expression of several methylation-related genes was also assessed in these samples (Figure 2B), revealing that the majority of these genes were downregulated in cattle-yak testicular samples relative to those in samples from both 5-years-old and 18-months-old yaks. Specifically, testicular *METTL14, YTHDF1, YTHDF2, RBM15, ZC3H13*, and *VIRMA* expression was significantly reduced in cattle-yaks relative to both analyzed yak samples, whereas testicular *WTAP, FTO, ALKBH5, YTHDC1*, and *YTHDC2* mRNA levels were significantly lower in cattle-yaks relative to 5-years-old yaks, although they did not differ significantly relative to 18-months-old yaks. Moreover, *METTL3* and *YTHDF3* expression was significantly increased in cattle-yaks relative to 18-months-old yaks, whereas they did not differ significantly from levels in 5-years-old yaks. The observed alterations in methylation-associated enzyme expression may underlie profound differences in the m^6^A methylation status of cattle-yak testicular tissues.

### Sequencing data quality control and reference genome comparisons

Next, the MeRIP-seq analysis of 6 samples was conducted (Supplementary Table S2), yielding 43.31 Gb of clean data. Each sample yielded 6.31–8.15 Gb of data, with Q30 base distributions of 94.83%-96.30%, with an average GC content of 56.70%. These reads were successfully aligned to a reference genome (LU_Bosgru_v3.0: http://ftp://ftp.ensembl.org/pub/release-99/fasta/bos_grunniens/dna/Bos_grunniens.LU_Bosgru_v3.0.dna_sm.toplevel.Fa.gz, with alignment rates of 76.01%-89.07% (Supplementary Table S3).

### Methylation peak detection and annotation

Next, the whole transcriptome m^6^A profile for cattle-yak testicular tissues were obtained by high-throughput sequencing. A total of 16,186 peaks (Supplementary Table S4) were detected, and the widths of most peaks was distributed between 1 bp and 1,000 bp (*n* = 8,059), for details of the numbers of methylation peaks in each width range (see Table 1). For further details regarding methylation peak width distributions, see Supplementary Figure S1A. As the predicted methylation sites varied among samples, they were next separated into five reliability-based subcategories: Non-reliable, Low, Moderate, High, and Very High (Supplementary Figure S1B). CHIPseeker-based annotation results revealed 1–18 m^6^A peaks per gene, with 54% of genes exhibiting only a single peak (Supplementary Table S5), while the *SYNE2* gene encoded on chromosome 11 exhibited the highest number of peaks (18 m^6^A peaks).

**Table 1:** The numbers of methylation peaks in each width range.

**Peaks width (bp)**	**TES (number)**
1–1,000	8,059
1,001–5,000	4,122
5,001–10,000	1,672
10,001–50,000	1,981
50,001–100,000	247
>100,000	105

**Figure 3:**
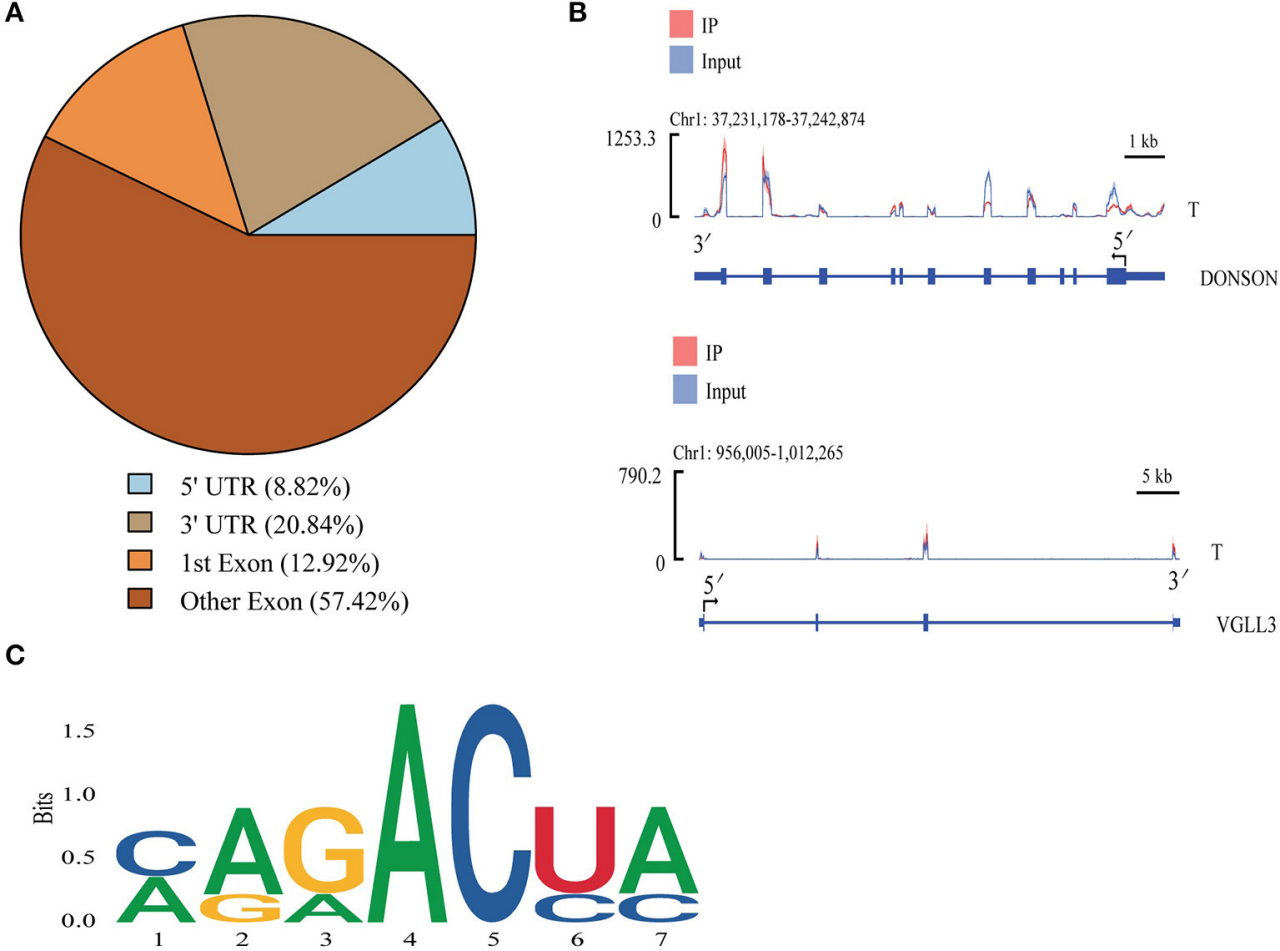
Cattle-yak methylation peak analyses. **(A)** mRNA methylation peak distributions. **(B)** IGV plots demonstrating peaks in the *VGLL3* and *DONSON* genes. **(C)** The motifs most commonly subjected to m^6^A modification in cattle-yak testicular samples.

Most peaks were concentrated in the exonic regions of associated genes, with progressively fewer peaks in the 3'-UTR and 5'-UTR regions (Figure 3A). To demonstrate representative patterns of m^6^A methylation, two model genes were selected for depiction. For the *VGLL3* mRNA, peaks were present in the CDS and 3'-UTR regions, while in the *DONSON* mRNA they were located in the 3'-UTR, CDS, and 5'-UTR regions (Figure 3B). As m^6^A modification is generally associated with the 5-RRACH-3 sequence (R = A or G; H = A, C or U) (38), the enrichment of m^6^A peaks in the common RRACH sequence was assessed, revealing comparable enrichment in these cattle-yak samples to previously reported data (Figure 3C). These results thus offer further credibility to these m^6^A peak data, supporting the regulatory role of a ubiquitous mechanism governing patterns of RNA methylation.

### Detection and annotation of differentially methylated peaks

**Figure 4:**
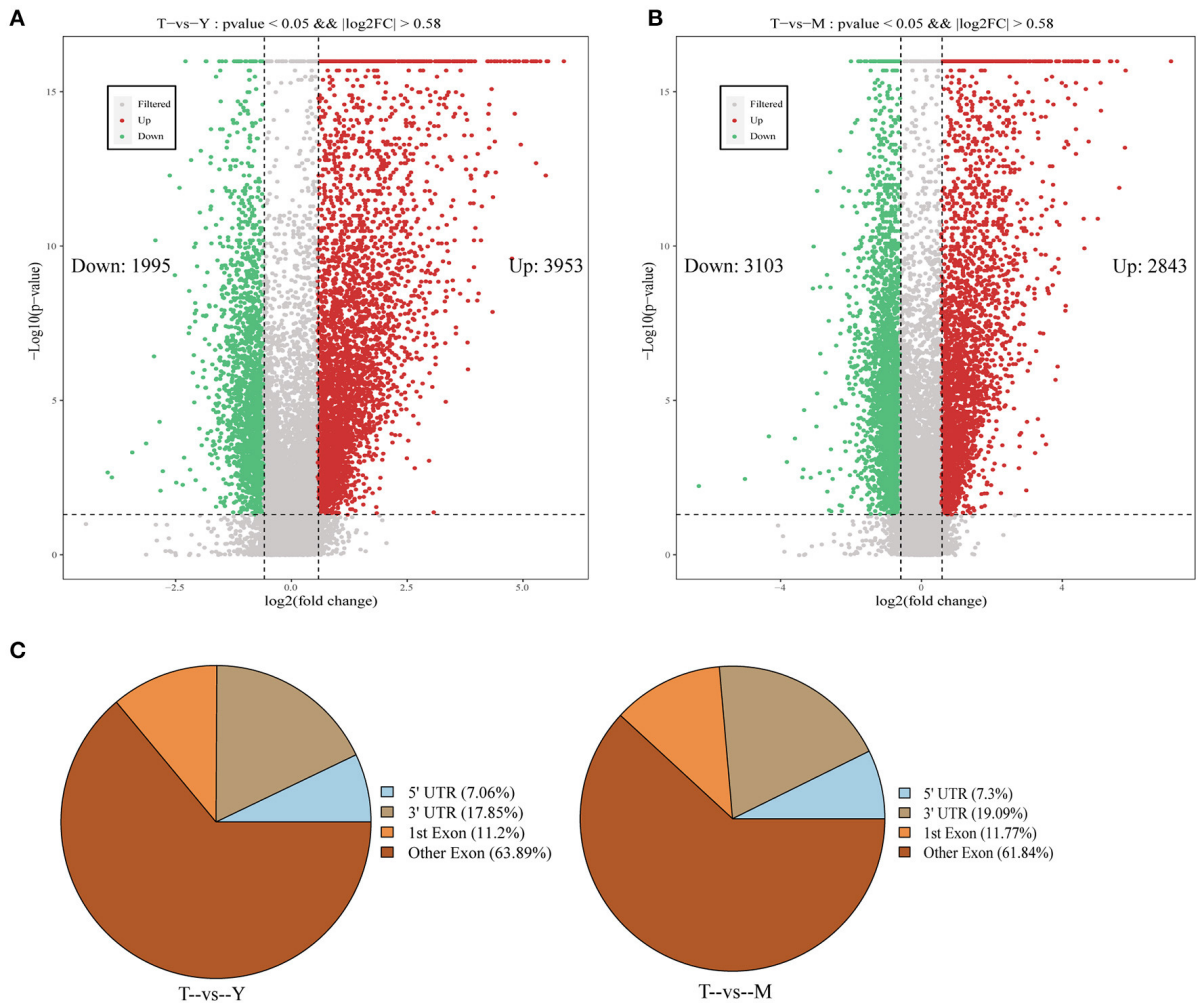
Identification and annotation of differentially methylated peaks. **(A,B)** Volcano plots demonstrating differentially methylated peak distributions. **(C)** Pie charts representing the annotation of DMPs associated with different gene regions.

Differentially methylated peaks (DMPs) were next analyzed with the MeTDiff software (screening criteria: diff. *p* ≤ 0.05; diff.fc ≥ 1.5), with 5,948 DMPs being identified for the T vs. Y comparison, of which 3,953 and 2,843 were upregulated and downregulated, respectively, in cattle-yak samples (Figure 4A). Similarly, 5,946 DMPs were identified for the T vs. M comparison, of which 3,103 and 2,843 were respectively upregulated and downregulated in cattle-yak samples (Figure 4B). DMP width distributions are presented in Supplementary Figure S1C. Distributions of detected DMPs on gene functional elements were further annotated, revealing the majority of these peaks to be concentrated in exonic regions, followed by the 3'-UTR and 5'-UTR regions (Figure 4C).

### Enrichment analyses of differentially methylated peak-associated genes

**Figure 5:**
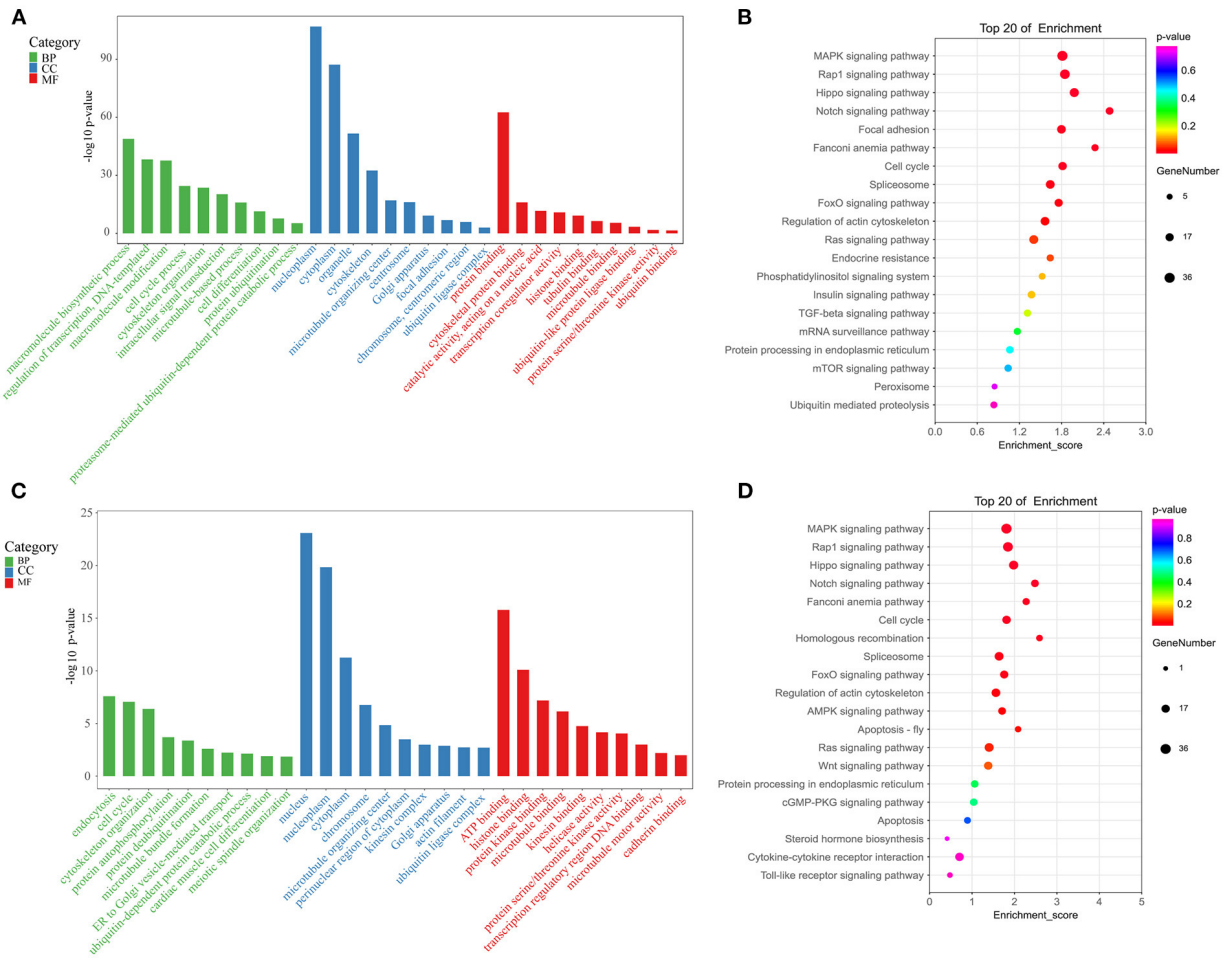
Enrichment analyses. **(A)** GO analyses for the T vs. Y group. **(B)** KEGG analyses for the T vs. Y group. **(C)** GO analyses for the T vs. M group. **(D)** KEGG analyses for the T vs. M group.

To gain potential insight into the effects of m^6^A methylation on gene expression and function in the testes of sterile male cattle-yaks, GO, and KEGG functional enrichment analyses of genes bearing DMPs were next conducted. GO analyses of the DMPs identified for the T vs. Y comparison revealed these genes to be enriched for molecular functions including cytoskeletal protein binding, tubulin binding, and ubiquitin-like protein ligase binding, cellular components including the microtubule organizing center, cytoskeleton, and Golgi apparatus, and biological processes including the cell cycle, cytoskeleton organization, and cell differentiation (Figure 5A). KEGG analyses for the T vs. Y comparison further indicated that DMP-associated genes were significantly enriched in the regulation of actin cytoskeleton, Notch signaling, cell cycle, and TGF-β signaling pathways (Figure 5B). GO analyses for the T vs. M group comparison similarly revealed DMPs to be enriched for molecular function terms including microtubule binding, kinesin binding, and protein serine/threonine kinase activity, cellular components including microtubule-organizing centers, actin filaments, and the ubiquitin ligase complex, and biological processes including meiotic spindle organization, ubiquitin-dependent protein catabolic process, and endocytosis (Figure 5C). KEGG analyses for the T vs. M comparison additionally indicated that these DMPs were associated with the homologous recombination, apoptosis, steroid hormone biosynthesis, and Fanconi anemia pathways (Figure 5B).

### RNA-seq-based differential gene enrichment analyses

**Figure 6:**
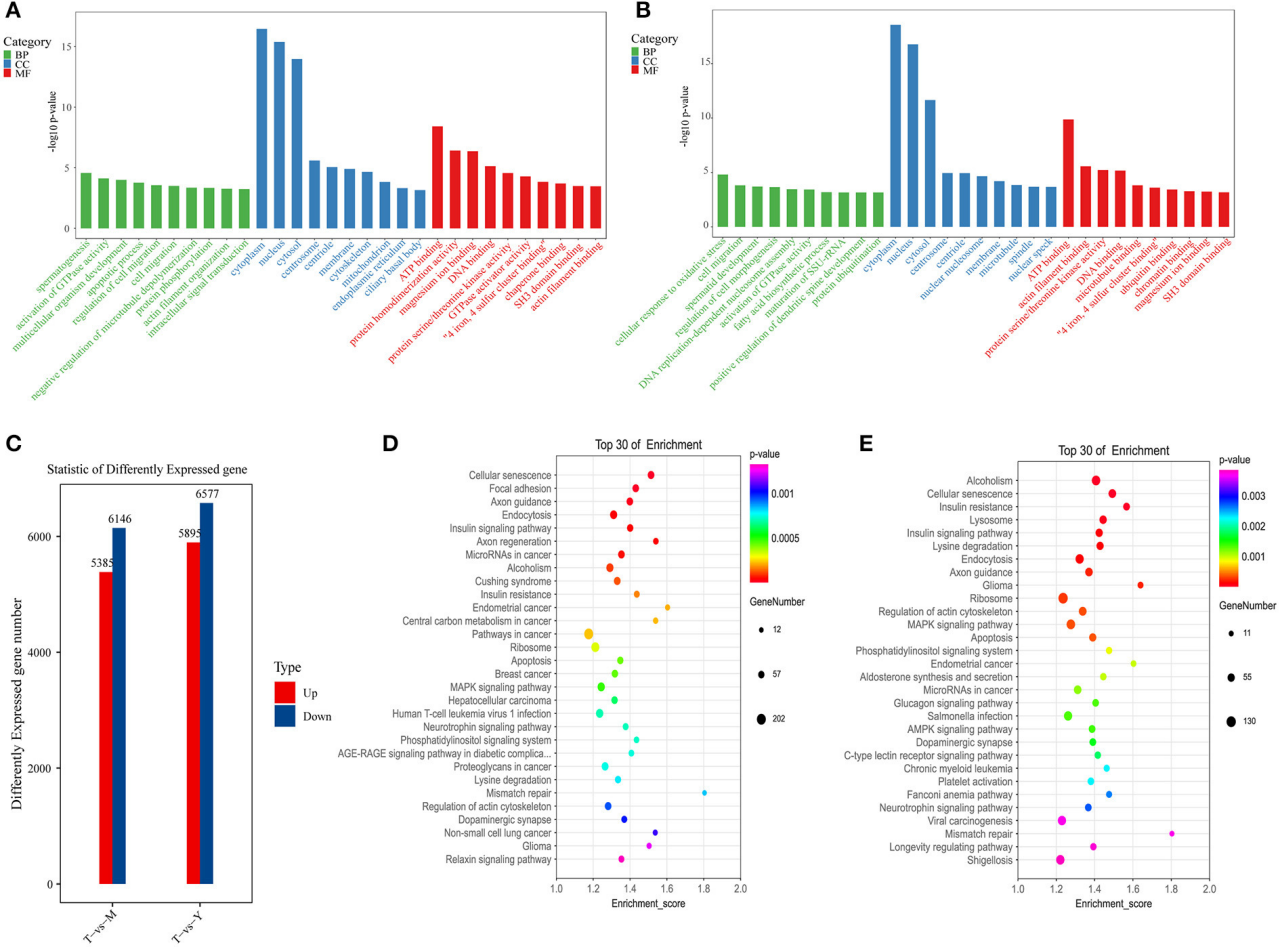
Functional enrichment analyses for DEGs. **(A)** GO analyses for the T vs. Y group. **(B)** KEGG analyses for the T vs. Y group. **(C)** DEG statistics. **(D)** GO analyses for the T vs. M group. **(E)** KEGG analyses for the T vs. M group.

Next, RNA-seq analyses were used to compare changes in mRNA expression in cattle-yak testis tissues (Supplementary Table S6). Relative to testis samples from 18-months-old yaks, cattle-yak samples exhibited 5,895 and 6,577 genes that were respectively up- and downregulated (*P* < 0.05, log2FC > 0.58). Relative to 5-years-old yak tissue samples, cattle-yak samples exhibited 5,385 and 6,146 genes that were up- and down-regulated, respectively (*P* < 0.05, log2FC > 0.58) (Figure 6C). GO analyses for differentially expressed genes (DEGs) identified for the T vs. Y group comparison revealed these genes to be enriched for molecular functions including actin filament binding, protein serine/threonine kinase activity, and ATP binding, cell components including the cytoskeleton, cytoplasm, and centrosome, and biological processes including spermatogenesis, actin filament organization, and intracellular signal transduction (Figure 6A). KEGG analyses for the T vs. Y comparison revealed these DEGs to be primarily enriched in the cellular senescence, regulation of actin cytoskeleton, and MAPK signaling pathways (Figure 6D). GO analyses for the T vs. M comparison revealed associated DEGs to be enriched for molecular function terms including ATP binding, actin filament binding, and protein serine/threonine kinase activity, cellular component terms including the cytoplasm, centrosome, and microtubules, and biological process terms including spermatid development, protein ubiquitination, and DNA replication-dependent nucleosome assembly (Figure 6B). Similarly, KEGG analyses for the T vs. M comparison revealed associated DEGs to primarily be enriched in the apoptosis, regulation of actin cytoskeleton, and Fanconi anemia pathways (Figure 6E).

### Combined analysis of genes differentially expressed and differentially methylated in yak and cattle-yak testis samples

**Figure 7:**
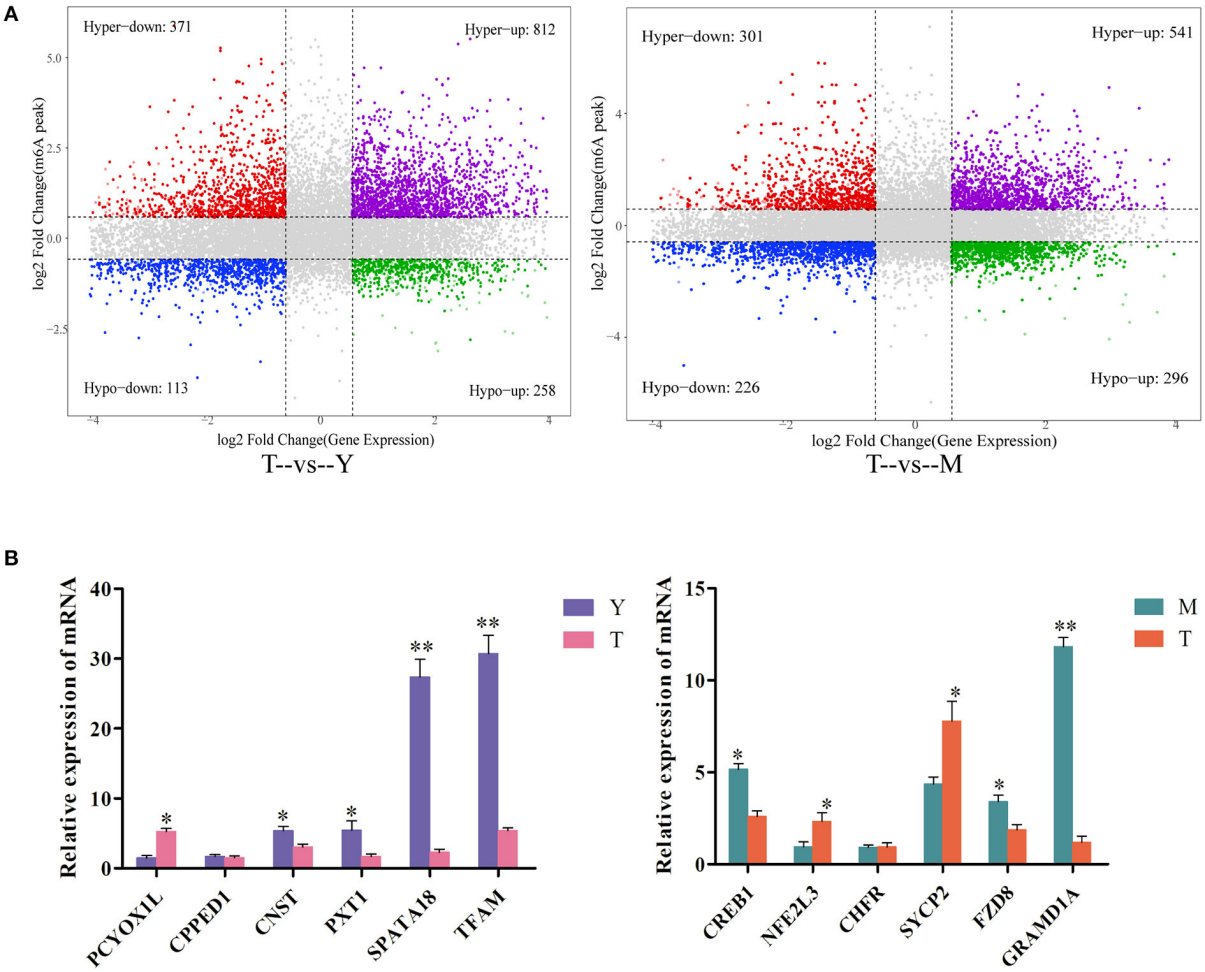
Combined analyses of differentially expressed and differentially methylated genes. **(A)** A combined four-quadrant scatterplot highlighting genes exhibiting significantly altered methylation and expression patterns when comparing the indicated samples, with gray dots corresponding to genes that did not exhibit any significant differences. **(B)** qPCR-based validation of DEGs identified *via* RNA-seq analyses. **P < *0.01*, *P<*0.05*.

To gain additional insight into the link between m^6^A methylation and mRNA expression, we further analyzed genes that were both differentially methylated (Log_2_ FC > 1, *P* < 0.05) and differentially expressed (log_2_ FC > 1, *P* < 0.05). In total, 1,554 genes for the T vs. Y comparison were significantly differentially methylated and expressed, of which 812 exhibited increased m^6^A peaks and increased mRNA expression, 371 exhibited increased m^6^A peaks and decreased mRNA expression, 113 exhibited reduced m^6^A peaks and increased mRNA expression, and 258 exhibited decreased m^6^A peaks and decreased mRNA expression (Supplementary Table S7). Moreover, 1,364 genes for the T vs. M comparison were differentially expressed and differentially methylated, of which 541 exhibited increased m^6^A peaks and increased mRNA expression, 301 exhibited increased m^6^A peaks and decreased mRNA expression, 226 exhibited decreased m^6^A peaks and increased mRNA expression, and 296 exhibited decreased m^6^A peaks and decreased mRNA expression (Supplementary Table S8). For further details regarding the link between m^6^A methylation status and mRNA expression levels (see Figure 7A).

Lastly, qPCR analyses were performed to confirm the expression of differentially methylated genes in testicular tissue samples from yaks and cattle-yaks. The detected expression patterns were consistent with RNA-seq results (Figure 7B), thus validating these transcriptomic analyses.

## Discussion

Distant hybridization is a term used to refer to hybridization between relatively distantly related species or genera (39), ultimately promoting the exchange of genes between different biotypes and thereby contributing to altered genotypic and phenotypic characteristics in the resultant offspring. Genotypic changes associated with distant hybridization can arise at the DNA and chromosomal levels, while at the phenotypic level heterosis can arise as a result of the integration of the relative advantages of the parents of a given offspring (40). Distant hybridization can support the development of new species (41–43), but male sterility is common among these hybrid offspring as a consequence of reproductive isolation (44, 45).

Research to date on hybridization-related male sterility has focused on crosses between chickens and quails, silver-black foxes and blue foxes, horses and donkeys, Muscovy ducks and domestic ducks, and yaks and cattle. Wodsedalek et al. (46) posited that male mule infertility following the hybridization of donkeys and horses may be attributable to differences in the numbers of chromosomes in these parental species, contributing to meiotic block and impaired spermatogenesis. Sun et al. (47) and Feng et al. (48), in contrast, found that hybrid offspring of quails and chickens exhibited the same number of chromosomes as both parental species (2*n* = 78), but that the chromosome morphology in these hybrid offspring differed significantly from that of either parent. Imbalances in gonadal enzyme ratios and abnormal hormone offspring in the hybrid offspring of Muscovy ducks and domestic ducks reportedly contribute to abnormalities in testicular anatomy and germ cell division in these animals, with an incomplete blood-testis barrier also potentially contributing to male sterility in this case (49). The prophase arrest of germ cell meiosis in the hybrid offspring of silver-black foxes and blue foxes, together with lower testosterone concentrations and increased prolactin and LH concentrations, can adversely impact spermatogenesis (50).

In prior studies, cattle-yak seminiferous tubules were reported to only harbor Sertoli cells and a limited number of spermatogonia, whereas they were devoid of spermatocytes or other identifiable germ cells. Moreover, these hybrid testis samples exhibited a spermatogenic cell monolayer that was loosely attached to the basement membrane, with increased spermatogonia apoptosis and the disruption of meiotic progression at the mid-pachytene (51). Consistently, H&E staining of cattle-yak testis samples in the present study revealed that only Sertoli cells and spermatogonia were present within the seminiferous tubules, confirming the accuracy of these sampling strategies. The anterior pituitary of cattle-yaks was largely devoid of basophilic cells, with severe segmentation of the nucleus of follicle-stimulating cells and limited numbers of secretory granules, thereby contributing to decreased FSH secretion, limiting seminiferous tubule development (52). Shah et al. (6) employed a STA-PUT approach to isolate spermatocytes and spermatogonia from cattle-yak testis, revealing the diameters of these cells to be significantly reduced relative to those of corresponding cells from yaks or cattle. Cai et al. (53) conducted an RNA-seq analysis assessing genes differentially expressed between yak and cattle-yak testicular tissues, leading to the identification of a link between *NLRP14* and *STRA8* upregulation and undifferentiated spermatogonia accumulation and apoptosis in cattle-yak testes. In contrast, the downregulation of the *SPP1, SPIN2B*, and *PIWIL1* genes was linked to impaired spermatogonia genomic integrity and cell cycle progression, with several other meiosis-assocaited genes also exhibiting some level of downregulation in this context. While many comprehensive analyses of these cattle-yak hybrids have thus been conducted to date, the specific mechanisms underlying male sterility in these animals has yet to be established.

Further qPCR-based quantitative analyses of methylation-associated enzymes in these animals revealed the majority to be expressed at lower levels in cattle-yak testes relative to testes from both 18-month-old and 5-year-old yaks. For example, *METTL14, YTHDF1*, and *RBM15* expression levels were significantly reduced in cattle-yak testicular tissues relative to tissues from 18-month-old and 5-year-old yaks, while *YTHDC2, FTO*, and *ALKBH5* levels were significantly reduced in cattle-yak samples relative to those from 5-year-old yaks. *ALKBH5*-mediated m^6^A modification can influence the stability and splicing of mRNAs with long 3'-UTRs in spermatocytes and round spermatids (54). As such, the m^6^A modification of *ALKBH5* is critical to the meiotic and haploid stages of the spermatogenic process (55). In mice, the knockout of *ALKBH5* can lead to the impairment of spermatogenesis and associated male sterility (56). *YTHDC2*-KO mice, in contrast, exhibit germ cells that fail to mature beyond the zygotic stage (57). The dual knockout of both *METTL3* and *METTL14* can suppress the translation of critical spermatogenesis-associated transcripts linked to m^6^A modification, contributing to further anaphase spermatogenesis abnormalities (18). Mutations in *FTO* are positively correlated with reductions in semen quality and may be linked to decreased male fertility (31). As such, enzymes associated with the process of m^6^A methylation are likely to be critical to the maintenance of normal male reproductive function, with decreases in methylation-related enzyme expression thus representing one potential cause of male cattle-yak sterility.

Methylation peaks in cattle-yak testis tissues were primarily concentrated in exonic regions, with lower levels in the 3'-UTR and 5'-UTR regions. Partial exon methylation peaks were largely concentrated at sites proximal to stop codons, in line with prior data from human and murine studies (58–60). Ke et al. (61) reported that the majority of m^6^A-modified residues are located near the final exon in a given transcript, providing a mechanism for 3'-UTR regulation. As such, overall m^6^A site distributions seem to be similar across different mammalian species. In prior studies, m^6^A peaks were shown to be enriched in regions harboring conserved RRACH motifs (58, 59), and the same was true for many of the m^6^A-associated sequences in the present study, in line with data from other studies of yeast, amphibians, plants, and mammals (26). Overall, these results provide support for the conservation of m^6^A methylation in mammals.

Functional enrichment analyses were conducted in an effort to explore the biological roles of DEGs and DMPs identified when comparing testis samples from cattle-yaks to those from 18-months-old or 5-year-old yaks. GO analyses for the T vs. Y group comparison revealed genes that were differentially expressed and differentially methylated to be associated with spermatogenesis-related GO terms including cytoskeleton and actin binding, and with the actin cytoskeleton regulation and MAPK signaling KEGG pathways. Moreover, GO analyses for the T vs. M group comparison revealed differentially expressed and differentially methylated genes to be associated with GO terms including protein ubiquitination, ubiquitin ligase complexes, ubiquitin-dependent protein catabolism, and endocytosis, and with the apoptosis and Fanconi anemia KEGG pathways. Fanconi anemia (FA) is a rare autosomal recessive genetic disorder in which patients experience progressive bone marrow failure associated with a range of congenital abnormalities, reduced fertility, and a higher risk of developing leukemia and head and neck squamous cell carcinoma (62, 63). Roughly 50% of women affected by FA are infertile, while fertility is rare among males diagnosed with FA (64, 65). In male FA patients, reduced fertility-related clinical symptoms include major reductions in spermatozoa with concomitant spermatozoa abnormalities, with Sertoli cell syndrome and non-obstructive azoospermia often being diagnosed in these individuals (66).

While methylation-associated enzymes are known to play a key role in the spermatogenic process and cattle-yak m^6^A transcriptional mapping has been conducted, how altered methylation levels ultimately contribute to cattle-yak male sterility has yet to be established. Here, analyses of cattle-yak testicular methylation profiles were performed at the whole-tissue level. However, the process of spermatogenesis is highly complex and relies upon synergistic interactions among multiple cell types. As such, further work will be necessary to establish the cell type-specific effects of RNA methylation in the context of male cattle-yak infertility. Future advances in single-cell sequencing technologies have the potential to offer new insight into the methylation of individual cells within cattle-yak testes, providing a foundation for future functional research exploring the link between m^6^A methylation and male cattle-yak sterility.

## Conclusion

In summary, analyses m^6^A methylation levels in cattle-yak testis tissues and associated gene enrichment analyses were performed in this study. The expression of related methylases in cattle-yak samples was significantly reduced relative to that in yaks both before and after sexual maturity. Enrichment analyses of differentially expressed genes and genes associated with differentially methylated peaks indicated that the differentially expressed genes identified when comparing cattle-yaks and pre-sexually mature yaks were mainly associated with spermatogenesis, including cytoskeleton and actin binding, and actin cytoskeleton regulation. In contrast, differentially expressed genes identified when comparing cattle-yaks and sexually mature yaks were primarily associated with protein ubiquitination, ubiquitin ligase complexes, ubiquitin-dependent protein catabolism, and endocytosis, as well as with the apoptosis and Fanconi anemia KEGG pathways.

## Data availability statement

The datasets presented in this study can be found in online repositories. The names of the repository/repositories and accession number(s) can be found in the article/Supplementary material.

## Ethics statement

All animal-related procedures were consistent with guidelines established by the China Council on Animal Care and the Ministry of Agriculture of the People's Republic of China. The Animal Care and Use Committee of the Lanzhou Institute of Husbandry and Pharmaceutical Sciences Chinese Academy of Agricultural Sciences approved all yak handling procedures for this study (Permit No: SYXK-2014-0002). Written informed consent was obtained from the owners for the participation of their animals in this study.

## Author contributions

Conceptualization: XG and JP. Data curation and writing—original draft: XW. Investigation: SG, MC, and YK. Software: YL and CL. Writing—Review and editing: LX, PB, and PY. Funding acquisition: XG. All authors contributed to the interpretation of the results, writing of the article, read, and agreed to the published version of the manuscript. All authors contributed to the article and approved the submitted version.

## Funding

This work was supported by the China Agriculture Research System of MOF and MARA (CARS-37) and the Innovation Project of Chinese Academy of Agricultural Sciences (25-LZIHPS-01).

## Conflict of interest

The authors declare that the research was conducted in the absence of any commercial or financial relationships that could be construed as a potential conflict of interest.

## Publisher's note

All claims expressed in this article are solely those of the authors and do not necessarily represent those of their affiliated organizations, or those of the publisher, the editors and the reviewers. Any product that may be evaluated in this article, or claim that may be made by its manufacturer, is not guaranteed or endorsed by the publisher.
