# Supplementary material for: Characterization of N6-methyladenosine in cattle-yak testis tissue
Source: Front Vet Sci. 2022 Aug 9;9:971515. doi: 10.3389/fvets.2022.971515 (PMC9395605; doi:10.3389/fvets.2022.971515)
Supplement: Supplementary file 1 [file Data_Sheet_1.docx]

**Characterization of N^6^-methyladenosine in Cattle-yak Testis Tissue**

**Xingdong Wang^1,2^, Jie Pei^1,2^, Shaoke Guo^1,2^, Mengli Cao^1,2^, Yandong Kang^1,2^, Lin Xiong^1,2^, Yongfu La^1,2^, Pengjia Bao^1,2^, Chunnian Liang^1,2^, Ping Yan^1,2^, Xian Guo^1,2^***

^1^Key Laboratory of Yak Breeding Engineering of Gansu Province, Lanzhou Institute of Husbandry and Pharmaceutical Sciences, Chinese Academy of Agricultural Sciences, Lanzhou, 730050, China

^2^Key Laboratory of Animal Genetics and Breeding on Tibetan Plateau, Ministry of Agriculture and Rural Affairs, Lanzhou, 730050, China

*** Correspondence:**Xian Guo
guoxian@caas.cn

**RT-qPCR**

To study the m6A status in cattle-yak testicular tissue after sexual maturation, the levels of RNA methylation-related genes such as *METTL3*, *METTL14*, *WTAP*, *FTO*, *ALKBH5*, *YTHDF1/2/3*, *YTHDC1/2*, *RBM15*, *VIRMA*, and *ZC3H13* were detected using qRT-PCR. The National Center for Biotechnology Information website was used for primer designing (Supplementary Table S9). RT-qPCR was performed using LightCycler® 96 Instrument (Roche, Beijing, China). The reaction volume was 20 µL, including 10 µL of 2× PrecisionPLUS Master Mix (Primerdesign), 1 µL of diluted cDNA (25 ng), 1 µL (300 nmol) of gene-specific forward and reverse primers each, and 7 µL of RNase/DNase free water. The standard PCR reaction conditions for all transcripts were as follows: 95°C (3 min), followed by 39 cycles of 95°C (10 s) and 55°C (30 s). *GAPDH* was used as the reference gene, and relative gene expression was examined using the 2^-ΔΔCT^ method (1). Each reaction was repeated in triplicate to obtain Ct values. Analysis of variance (ANOVA) was used to analyze differences in the expression of methylation-related enzymes.

**MeRIP-seq and RNA-seq and sequencing data analysis**

First, the quality and quantity of total RNA were estimated using Bioanalyzer 2100 (Agilent, CA, USA) and NanoDrop 2000 (Thermo Scientific), both with an RIN number > 7.0. More than 100 μg of total RNA was used for mRNA isolation via an mRNA Purification Kit (Ambion Dynabeads mRNA Purification Kit). Subsequently, poly (A) mRNA fractions were created using divalent cations and a thermocycler (RNA solution kept at 94°C for exactly 5), incubated with m^6^A-Dynabeads (Anti-m^6^A, Synaptic Systems, Cat. No 202003) in an m^6^A-binding buffer (50 mM Tris-HCl pH 7.4, 150 mM NaCl_2_, 1% NP-40, 2 mM EDTA), and allowed to bind to the beads. Following this, the m^6^A-Dynabeads were washed and m^6^A-positive RNA was eluted. The RIP was extracted and cleaned, and finally, m^6^A “enriched RNA” was collected. We used 100 ng of RNA (100 ng of input and 100 ng of post m^6^A-IP positive fraction) for library construction with the Illumina TrueSeq Stranded mRNA platform. Finally, paired-end sequencing was performed on an Illumina HiSeq X10 system at OE Biotech Co., Ltd. (Shanghai, China) using manufacturer’s instructions. The data was submitted to the GENE EXPRESSION OMNIBUS (GEO) database (accession number GSE205649).

Raw reads obtained from RNA sequences were subjected to statistical analysis and quality control (Supplementary table S1). Raw data (raw reads) in the fastq format were processed using the Trimmomatic (2) software, and reads with adapter sequences or poly-N sequences as well as low-quality reads were removed to obtain clean data. Following this, 250,000 paired reads were randomly extracted from the clean data, and blastn was used with the NT database (ftp://fp.ncbi.nih.gov/blast/db) to examine sequence alignment with the reads. The most high-quality results with an e value < 1e-10 and coverage > 80% were selected. Meanwhile, the SortMeRNA (3) software was used for removing ribosomal RNA reads. The remaining clean reads were mapped to the reference genome, LU_Bosgru_v3.0 (ftp://ftp.ensembl.org/pub/release-99/fasta/bos_grunniens/dna/Bos_grunniens.LU_Bosgru_v3.0.dna_sm.toplevel. Fa. gz) for sequence alignment using HISAT2 (4) to obtain position information on the reference genome as well as specific sequence characteristic information from the sequenced samples. Default parameters were used, and unique reads showing high mapping quality were retained. The Guitar (5) R package and deeptools (6) software were used to evaluate m6A-seq data quality in order to assess the quality of MeRIP-seq data.

m6A-enriched peaks were detected in each ample using the MeTDiff peak calling software (screening criteria: p <= 0.05; fc >= 1.5) and the options FRAGMENT_LENGTH=200, PEAK_CUTOFF_PVALUE=0.01, and PEAK_CUTOFF_FDR=0.05) (5). The number, width, and distribution of peaks were statistically analyzed, and the corresponding input sample was used as the control. The identified peaks were annotated based on their intersection with the gene architecture using ChIPseeker (7). Finally, differential analysis of m6A-seq data was performed by comparing data from yak and cattle-yak. This was done using MeTDiff (screening criteria: diff.p <= 0.05; diff.fc >= 1.5) with the following parameters: FRAGMENT_LENGTH=200, PEAK_CUTOFF_PVALUE=0.01, DIFF_PEAK_CUTOFF_FDR=0.05, and PEAK_CUTOFF_FDR=0.05). The differential peaks were again annotated using ChIPseeker.

Gene ontology (GO) enrichment and Kyoto Encyclopedia of Genes and Genomes (KEGG) pathway enrichment analyses of the identified peaks and differential peaks were performed using R based on hypergeometric distribution. Sequence motifs were identified using MEME (8) and DREME (9) and annotated using Tomtom software.

**REFERENCES**

1. Jozefczuk J, Adjaye J. Quantitative Real-Time Pcr-Based Analysis of Gene Expression. *Methods in enzymology* (2011) 500:99-109. doi: 10.1016/b978-0-12-385118-5.00006-2.

2. Bolger A, Lohse M, Usadel B. Trimmomatic: A Flexible Trimmer for Illumina Sequence Data. *Bioinformatics (Oxford, England)* (2014) 30:2114-20. doi: 10.1093/bioinformatics/btu170.

3. Kopylova E, Noé L, Touzet H. Sortmerna: Fast and Accurate Filtering of Ribosomal Rnas in Metatranscriptomic Data. *Bioinformatics (Oxford, England)* (2012) 28:3211-7. doi: 10.1093/bioinformatics/bts611.

4. Kim D, Langmead B, Salzberg S. Hisat: A Fast Spliced Aligner with Low Memory Requirements. *Nature methods* (2015) 12:357-60. doi: 10.1038/nmeth.3317.

5. Cui X, Wei Z, Zhang L, Liu H, Sun L, Zhang S, et al. Guitar: An R/Bioconductor Package for Gene Annotation Guided Transcriptomic Analysis of Rna-Related Genomic Features. *BioMed research international* (2016) 2016:8367534. doi: 10.1155/2016/8367534.

6. Ramírez F, Dündar F, Diehl S, Grüning B, Manke T. Deeptools: A Flexible Platform for Exploring Deep-Sequencing Data. *Nucleic acids research* (2014) 42:W187-91. doi: 10.1093/nar/gku365.

7. Yu G, Wang L, He Q. Chipseeker: An R/Bioconductor Package for Chip Peak Annotation, Comparison and Visualization. *Bioinformatics (Oxford, England)* (2015) 31:2382-3. doi: 10.1093/bioinformatics/btv145.

8. Bailey T, Boden M, Buske F, Frith M, Grant C, Clementi L, et al. Meme Suite: Tools for Motif Discovery and Searching. *Nucleic acids research* (2009) 37:W202-8. doi: 10.1093/nar/gkp335.

9. Schulz M, Devanny W, Gitter A, Zhong S, Ernst J, Bar-Joseph Z. Drem 2.0: Improved Reconstruction of Dynamic Regulatory Networks from Time-Series Expression Data. *BMC systems biology* (2012) 6:104. doi: 10.1186/1752-0509-6-104.
