# Supplementary material for: Characterization of N6-methyladenosine in cattle-yak testis tissue
Source: Front Vet Sci. 2022 Aug 9;9:971515. doi: 10.3389/fvets.2022.971515 (PMC9395605; doi:10.3389/fvets.2022.971515)
Supplement: Supplementary file 2 [file Table_1.docx]

Supplementary Table S1. Primer sequence.

| **Gene** | **Primer sequences (5' to 3')** | **TM/ ^o^C** |
| --- | --- | --- |
| METTL3 | F: TAGCCAAGGAGCCAACCAAG  R: TCTTGAACTTGAGCCCGACC | 57 |
| METTL14 | F: TCTGGGGAAGGATTGGACCT  R: CCCGTCTGTGCTACGCTTTA | 57 |
| WTAP | F: GTCTGGATTTCACAGGGAGGG  R: CAGGTTTCTCCTCTTGCGGG | 59 |
| FTO | F: GCACAAGCACGGCTGTTTAT  R: TTGACAGGCGGCAGCTATTT | 55 |
| ALKBH5 | F: ACGAGATTAGATGCACCCCG  R: CTGTTGTTTCCCGACAGACG | 57 |
| YTHDF1 | F: GTCACCACCTCTGCAGTCAA  R: TGTTTAATAGGTGGCGGGGG | 57 |
| YTHDF2 | F: CTGCGAAACAGCAACCCAAG  R: TGCCACAGGACCCTTGTTATC | 57 |
| YTHDF3 | F: CAGAGGAAACAGGCGAAGAA  R: CTGCTTCCCCAAGCGAATATG | 57 |
| YTHDC1 | F: GAAGCTCTGCATCGGAGTCA  R: CTTTTCGGACAGCACGAACG | 57 |
| YTHDC2 | F: AGTGGAAGTGATCTCAGTGCAG  R: GTGCATTTTCAGCAAACCGC | 56 |
| RBM15 | F: GAGTTTGACCGGTTTGGCAC  R: TGAGTTAACGGCAGAGGCTG | 57 |
| ZC3H13 | F: CCCCAGGGATTCTCGGTCTA  R: CTACTTCTGCCCATCCGGTC | 59.5 |
| VIRMA | F: ACTACTGCCTTGCGTGTTCT  R: AGCGAGCCATCGAAATGGTA | 55.4 |
| CREB1 | F: AACCAGCAGAGTGGAGATGC | 57 |
|  | R: GTTGAAATCTGAACTGTCTGGACT |  |
| NFE2L3 | F: GAACCGACGGCTCAAGTGTC | 57 |
|  | R: CCAGTGAGACTCCCTCCAGTA |  |
| CHFR | F: GTGAGTCCTCGGACGTTAGC | 57 |
|  | R: GCACAGCCCCAGTATAGGTG |  |
| SYCP2 | F: TGGCTTGTCAGTGGTTCTCAA | 57 |
|  | R: TGACTGCTTCCCACTGATGAT |  |
| FZD8 | F: GGCCCATCATATTCCTCTCGG | 57 |
|  | R: CCAGCACGGCGATAGACTT |  |
| GRAMD1A | F: AGAACGCGCTGCTTGAAAAG | 57 |
|  | R: TACATCGCCCACATCCTTGG |  |
| PCYOX1L | F: CTCCACATGCAGGGTTTCGT | 57 |
|  | R: ACACCCGAGAAGGCATAACC |  |
| CPPED1 | F: GAACAGGAGATCCGTCTGGC | 57 |
|  | R: GAACGAGGAACAGGACACCC |  |
| CNST | F: AGTGACACATGCCGTTAGTGA | 57 |
|  | R: TTCCCACTGTTCTTGAATGGC |  |
| PXT1 | F: CTTCTCAGCCCAAGGAGGAT | 57 |
|  | R: TCCCCAATGTTTCTCAGGCG |  |
| SPATA18 | F: GCCATATCCCTTTTGGCTGC | 57 |
|  | R: TCGTCCTTCAGTGTTCGGAG |  |
| TFAM | F: CGACTGCGCTCTCCCTTTAG | 57 |
|  | R: CTGCCAGTCTGCCCTGTAAG |  |
| GAPDH | F: TCACCAGGGCTGCTTTTA | 60 |
|  | R: CTGTGCCGTTGAACTTGC |  |
