# Supplementary figures and images for: Characterization of N6-methyladenosine in cattle-yak testis tissue
Source: Front Vet Sci. 2022 Aug 9;9:971515. doi: 10.3389/fvets.2022.971515 (PMC9395605; doi:10.3389/fvets.2022.971515)

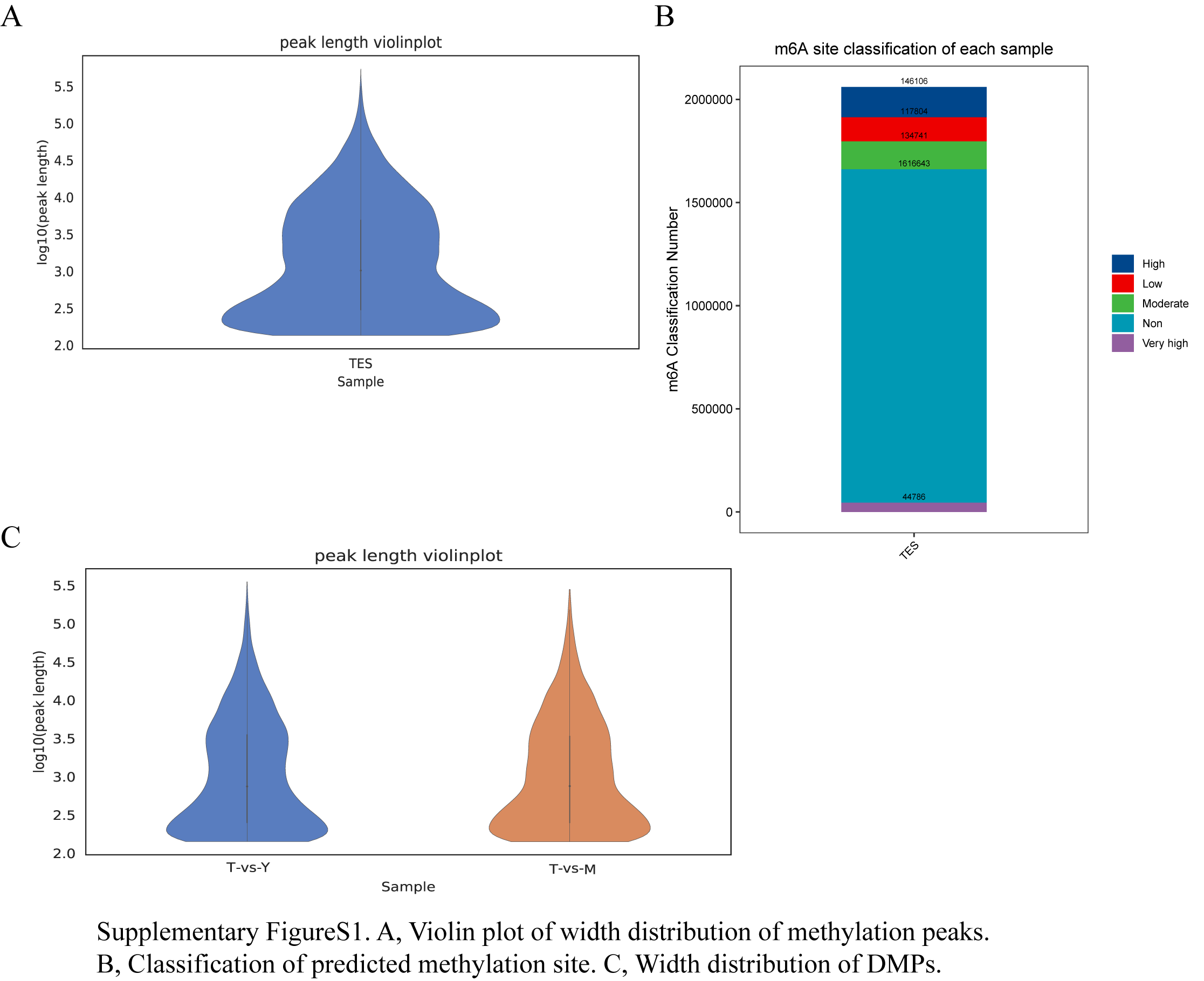

Supplement: Supplementary file 10 [file Image_1.tif]
